# Delivering and evaluating a scalable training model for strengthening family medicine in resource-limited environments: the Gaza experience. A mixed-methods evaluation

**DOI:** 10.3399/bjgpopen19X101647

**Published:** 2019-06-12

**Authors:** Ben Lovell, Ram Dhillon, Ali Khader, Akihiro Seita, Akiko Kitamura, Ghada Al-Jadba, Salman Rawaf, Roger Newson

**Affiliations:** 1 Consultant Physician & Postgraduate Lead in Acute Medicine, University College London Hospital, London, UK; 2 Consultant Surgeon, London North West University Healthcare NHS Trust, UK & Director of Postgraduate Medical Education, Rila Institute of Health Sciences, London, UK; 3 Health Policy & Planning Officer, UNRWA HQ, Amman, Jordan; 4 Director, Department of Health, UNRWA, Amman, Jordan; 5 Public Health Specialist / Epidemiologist, UNRWA, Amman, Jordan; 6 Chief of Field Health Programme, UNRWA, Gaza, Palestine; 7 Director, WHO Collaborating Centre, Imperial College, London, UK; 8 Research Associate, Department of Primary Care and Public Health, Imperial College, London, UK

**Keywords:** Scalable postgraduate medical training, postgraduate training, primary health care, resource-limited, education, community medicine

## Abstract

**Background:**

Since 2007, Gaza Palestine has been subject to blockade affecting over 1.9 million people. This denies health professionals access to continuing professional development (CPD). In Gaza, family physicians are scarce, and their level of training does not meet the needs of United Nations Relief and Works Agency’s (UNRWA) Family Health Team (FHT) model for better population health.

**Aim:**

This study sought to develop a postgraduate training programme for Gazan doctors via a Diploma in Family Medicine (FM PG), and evaluate its impact on physicians and patients.

**Design & setting:**

A mixed-methods evaluation of a postgraduate diploma in Gaza Palestine.

**Method:**

The programme was delivered over 1 year, to 15 primary care doctors. The impact was evaluated through focus group discussions and patient feedback questionnaire survey comparing FM PG graduate doctors and doctors without the FM PG Diploma.

**Results:**

All participating doctors graduated successfully and found the experience extremely positive. Trainees felt that the Diploma helped them take more individualised approach to patients; have a better understanding of psychosocial elements affecting patient health; feel more inclined towards team-working and collaborative approaches to health care; and more insight into non-verbal communication such as active listening and tactile gestures. Statistical analysis of patients’ feedback showed significantly improved patient-reported outcomes and satisfaction when treated by course diplomates compared to non-diplomates.

**Conclusion:**

Where there are limited training opportunities, investment in a structured postgraduate diploma training programme can improve quality of health service delivery. UNRWA’s experience in Gaza demonstrates the value of a scalable model in resource-limited settings.

## How this fits in

Where there are limited training opportunities, investment in a structured diploma programme can improve quality of healthcare delivery. UNRWA’s experience demonstrates the value of a scalable model in resource-limited settings. The authors urge other global health practitioners to examine these efforts and the progress in Gaza, and use this diploma programme as a template for future endeavours for international CPD.

## Introduction

### UNRWA

UNRWA was established in 1949, in the aftermath of the Arab–Israeli conflict, specifically to assist 750 000 displaced Palestinian refugees with humanitarian relief and welfare. It was envisioned that UNRWA would operate for 5 years; however, due to the absence of resolution to the Arab–Israeli crisis, the United Nations (UN) has continually renewed UNRWA’s mandate. UNRWA currently provides education, health and social services, and emergency assistance to over 5 million Palestinian refugees, including 2.2 million in the Palestinian territories of Gaza Strip and West Bank.^1^

**Figure 1.**
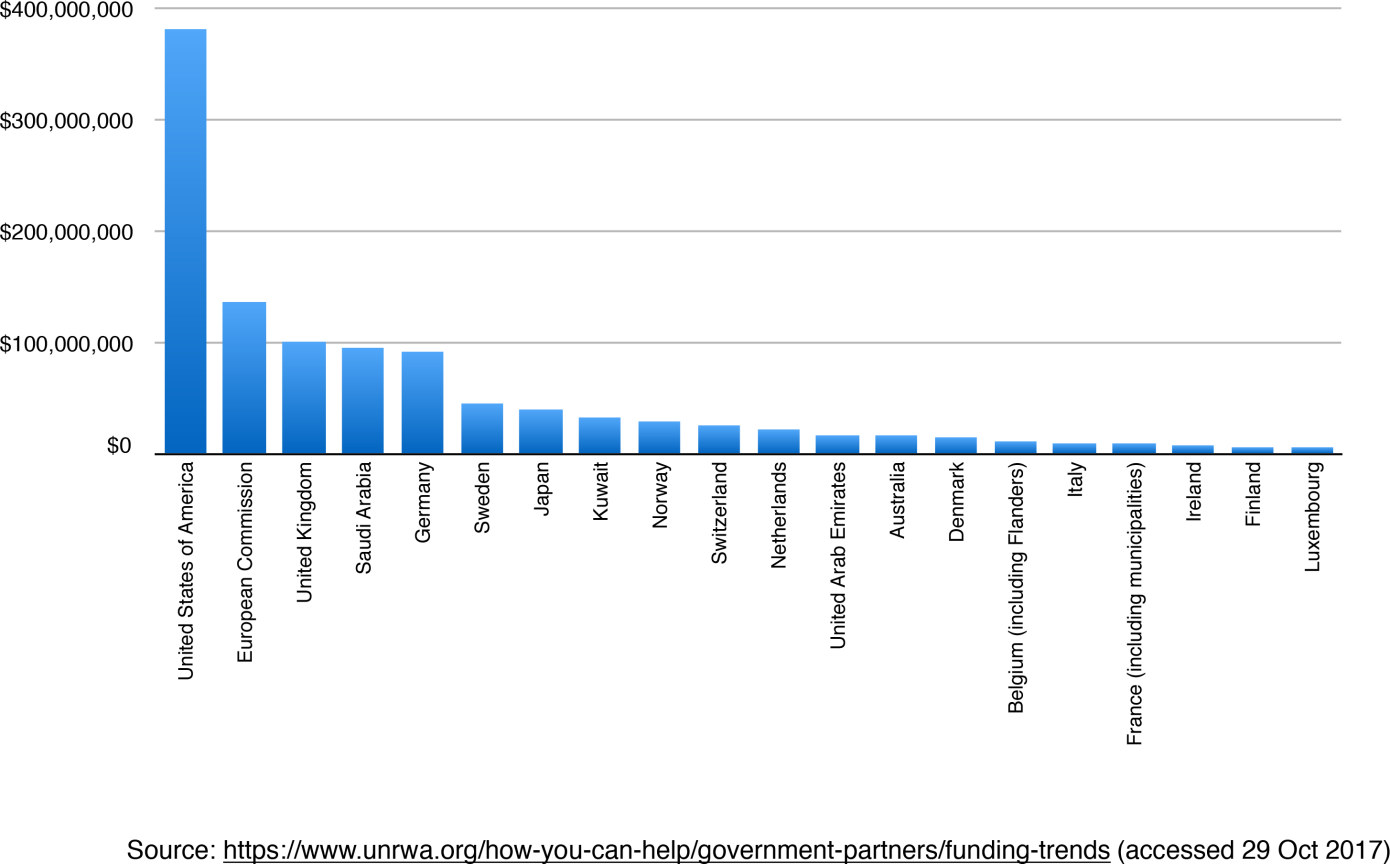
Top 20 government donors to United Nations Relief and Works Agency (UNRWA) in 2015, with the amount donated in US dollars

UNRWA is almost entirely funded by voluntary governmental contributions from UN member states (Figure 1). Smaller donations come from non-governmental organisations and charities. Due to the lack of a sustainable solution for Palestinian refugees, there are currently no plans to scale down this ‘temporary’ agency’s work in the Middle East.

### Gaza

Gaza Strip is a Palestinian territory in the Eastern Mediterranean, covering 350 km^2^. It is the world’s 20th most population-dense territory, with an estimated population of 1.9 million.^2^ Since 2007, Gaza has been subject to a total military blockade and movement across the border in either direction remains severely restricted.

Due to years of conflict and blockade, 80% of the population of Gaza is dependent on international assistance, and the number of Palestinian refugees relying on UNRWA for food aid has increased from fewer than 80 000 in 2000 to almost 1 million today.^3^

Health services are provided through a network of 22 health centres (11 health centres inside camps and 11 outside camps). These health centres are run by 963 health care workers composed of GPs, specialists, and nurses. The ratio of doctors per 100 000 people is 11.3; for nurses, it is 21.4.

The average number of medical consultations per doctor per day was 77 in 2017, compared to 82 in 2016, and 87 in 2015. The introduction of the FHT approach in 2011 has begun to help reduce the workload, mainly through the shifting of some tasks from medical officers to nurses, such as authority to approve monthly refills of medicines.

### Developing family practice model and scaling up family medicine training

Following the launch of UNRWA’s FHT model^4^ for GPs in 2012, it quickly became evident that UNRWA required the up-skilling of primary care doctors in the region. CPD opportunities for Gazan doctors are virtually non-existent.^5^ There are no congresses or national meetings for sharing knowledge, updating skills, or networking, and Gazan medics are not permitted to leave Gaza to attend international conferences.

Hence, a decision was taken to introduce a formal and structured training programme for UNRWA’s primary care doctors. The purpose of this article is to describe how such a programme was delivered and to evaluate its impact.

## Method

### The FM PG Diploma

**Figure 2.**
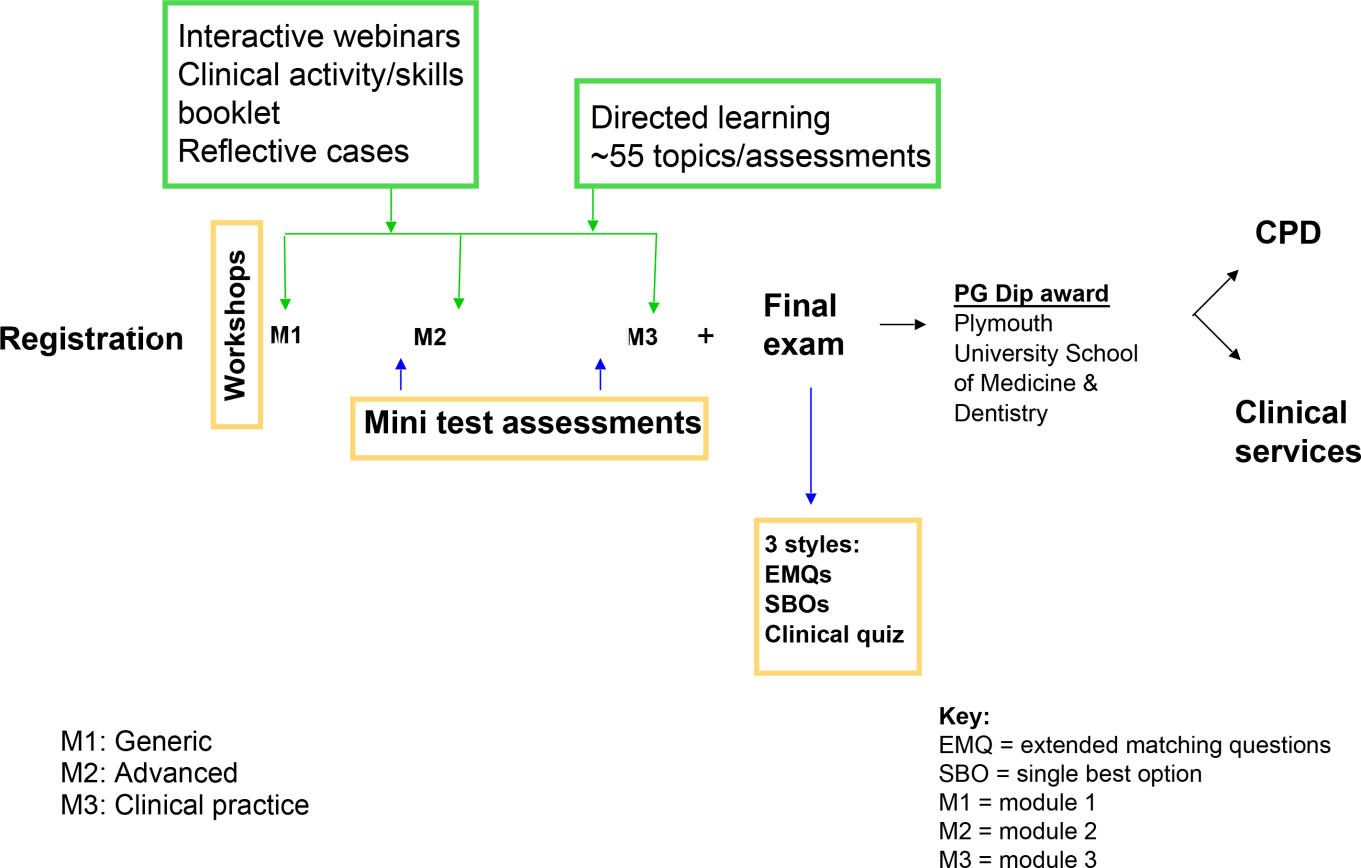
Structure of the Postgraduate Diploma in Family Medicine

In 2015, UNRWA approached the Rila Institute of Health Sciences in London UK through Imperial College London’s World Health Organization (WHO) Collaborating Centre for Public Health Education and Training for the purpose of devising a training programme for the family doctors working in Gaza. These family doctors had received formal specialty training in family medicine following graduation. The syllabus was developed in alignment with the core curriculum of the Royal College of General Practitioners. This curriculum was delivered through a blended training and work-based format, with a dedicated e-learning platform, direct face-to-face contact, interactive webinars, online core reading, formative assessments, self-assessed clinical case studies, and a practical skills module using video clips and clinical videos. There was a formal final summative examination (Figure 2).

### Recruitment of family doctors

The inaugural FM PG Diploma commenced in July 2015. The course content, purpose, and nature was communicated to all primary care doctors working with UNRWA in Gaza (*n* = 155). As the number of applicants exceeded the required number for the course, a transparent selection process was conducted. Applicants sat a pre-selection test, prepared by Rila and UNRWA, and doctors with the highest score were invited for enrolment into the programme at the Medical School at Al-Azhar in Gaza.

### Evaluation

Both quantitative and qualitative methods were employed to evaluate the impact of the PG FM Diploma, by way of patients’ surveys and feedback from doctors.

Two convenience samples of patients receiving care at UNRWA primary care centres in Gaza were generated. The first sample (*n* = 49) comprised patients receiving care from two family doctors who had successfully achieved the Diploma. The second sample (*n* = 47) had received care from two Gazan doctors who had not undertaken the Diploma. This evaluation took place 4 months after the completion of the Diploma.

The patient interviews were conducted by UNRWA staff who were blinded to the doctor’s participation (or otherwise) in the programme. The Consultation and Relational Empathy (CARE) Measure was used by patients to quantitively score their physicians after their consultations. The CARE measure is a validated and reliable tool of consultation quality,^6^ which was developed to measure physician empathy from the patient perspective.^7^ One further question was added to the 10 standard questions in the CARE measure: ‘How good was your doctor overall?’.

All analyses compared the results between the diplomates and the non-diplomates. Each of the questions was measured on a 7-point Likert scale from ‘poor’ to ‘outstanding’. To allow for the ordinal nature of the outcomes, the comparisons between groups were made using Somers' *D*, which is the parameter corresponding to the Mann-Whitney test, on a scale from -1 to 1, estimated with confidence intervals (CIs) and *P* values calculated using Fisher's *z*-transformation.^8^ Also reported are *q* values (or adjusted *P* values), calculated using the Simes-Benjamini-Hochberg procedure,^9^ indicating the significance of the group differences adjusted for the fact that there are 11 multiple comparisons.

The second method of evaluation focused on qualitative feedback was from the family doctors, following completion of the PG FM Diploma. Three individual focus groups took place, each comprising five diplomates; all 15 diplomates participated. These focus groups were led by three doctors from UNRWA and the Rila Institute, and were designed as semi-structured interviews, anchored to three key questions:

1. What did you hope to gain from the diploma?
2. What do you think you gained from the diploma?
3. How has your practice changed during the diploma? Do you have any examples?

The transcripts of the interviews were thematically sorted into individual codes by the interviewers in accordance with qualitative analysis principles. The codes were then cross-referenced for recurrent themes and ideas, and sorted into overarching categories. Analysis was carried out individually, and then the codes were discussed as a group until a consensus was reached. After the participation of 15 diplomates, the interviewers unanimously agreed that theoretical saturation had been attained, and no further interview sessions were arranged.

## Results

### Patient surveys

**Figure 3.**
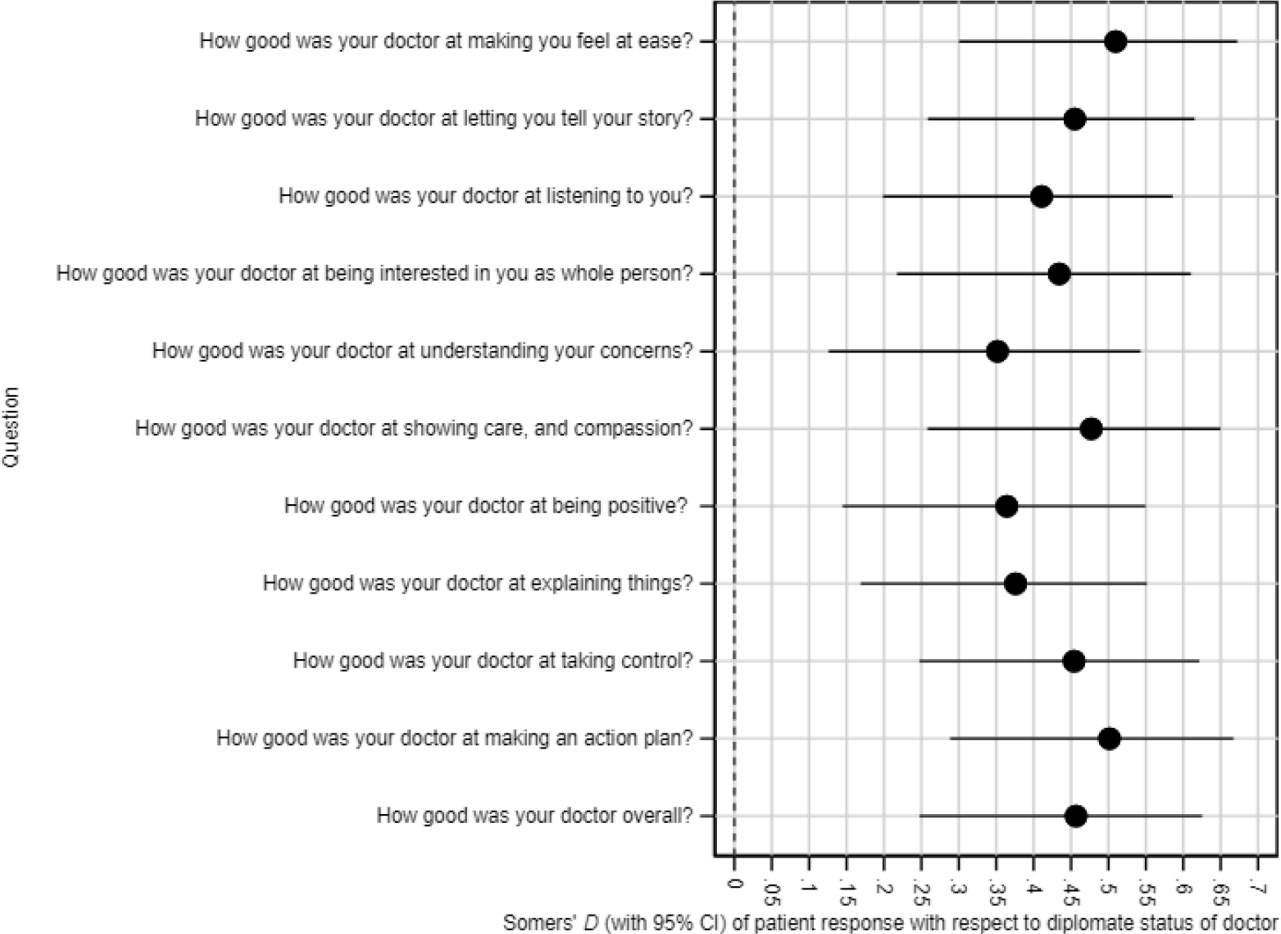
Confidence interval plot. Somers’ D of patient response with respect to diplomate status of doctor

Table 1 shows the Somers' *D* estimates for each of the 11 patient satisfaction scores with respect to diplomate status of the doctor, together with CIs, *P* values, and *q* values. Somers' *D* is a difference between two probabilities, namely the probability of concordance (the event that a random patient of a diplomate doctor is more satisfied than a random patient of a non-diplomate doctor) and the probability of discordance (the event that a random patient of a non-diplomate doctor is more satisfied than a random patient of a diplomate doctor). For instance, the Somers' *D* of the final question ‘How good was your doctor overall?’ with respect to diplomate status is 0.457 (95% CI = 0.248 to 0.625). This means that, if a random patient in the sample was sub-sampled from each treatment group (patients of a diplomate or non-diplomate doctor), then the patient of a diplomate doctor is 45.7% more likely than the patient of a non-diplomate doctor to be the more satisfied of the two patients. In the population of patients from which this sample was drawn, one can be 95% confident that the difference between these probabilities is between 24.8% more likely and 62.5% more likely. The Somers' *D* estimates for the other 10 satisfaction scores tell a similar story. The *P* values indicate that differences of this size are very unlikely to arise from sampling error, and the *q* values show that such a list of differences is still unlikely, allowing for the fact that patient satisfaction has been measured in 11 different ways using 11 different questions. The full data are available from the author on request. For completeness, the CI plot is provided in Figure 3.

**Table 1.** Somers' *D* of patient response with respect to diplomate status of doctor

Question	Patients, *n*	Somers' *D*	95% CI	*Q* value
How good was your doctor at making you feel at ease?	96	0.51	0.300 to 0.672	0.0002
How good was your doctor at letting you tell your story?	96	0.45	0.259 to 0.615	0.0002
How good was your doctor at listening to you?	96	0.41	0.199 to 0.586	0.0005
How good was your doctor at being interested in you as whole person?	96	0.43	0.217 to 0.610	0.0004
How good was your doctor at understanding your concerns?	96	0.35	0.126 to 0.543	0.003
How good was your doctor at showing care and compassion?	96	0.48	0.258 to 0.650	0.0002
How good was your doctor at being positive?	96	0.36	0.145 to 0.550	0.002
How good was your doctor at explaining things?	96	0.38	0.169 to 0.551	0.0009
How good was your doctor at helping you take control?	96	0.45	0.247 to 0.621	0.0002
How good was your doctor at making an action plan with you?	96	0.50	0.288 to 0.667	0.0002
How good was your doctor overall?	96	0.46	0.248 to 0.625	0.0002

CI = confidence intervals.

### Physicians’ feedback

Illustrative quotes and excerpts from the focus groups discussions with family doctors with the PG FM Diploma are available from the author on request. Analyses of these data yield several insights. The learners rarely mentioned knowledge acquisition as a primary motivating factor for undertaking the diploma. Instead, most focused on improving their clinical reasoning abilities, providing holistic and equitable service to their communities using guidelines and evidence-based approaches, and improving their confidence in their own abilities.

The self-reflections of new diploma graduates reveal a strong focus on examining their own practice, understanding the importance of taking a ‘slowed down approach’ to individual patient care, ways to improve their non-verbal communication such as active listening and tactile gestures, and the benefits of enhanced teamworking and burden-sharing within clinical teams. Individual quotes from the focus groups include the following:

*‘I have learned to talk to patients more often’* (Diploma holder)

*‘I have learned to make eye contact and use body language to communicate better with patients.’* (Diploma holder)

*’… learned the importance of teamwork, involving and supporting all of my colleagues, helping them when they are particularly busy*.’ (Diploma holder)

Responders also spoke of delivering better individualised, patient-centred care, seeing the patient as a person rather than a clinical conundrum or a task to solve.

There is evidence that diplomates are prepared to take deeper dives into the presenting history to detect more complex biopsychosocial phenomena within the family dynamic rather than drawing conclusions based on a superficial examination:

*‘ … identifying a case of Tourettes syndrome in a child who was being beaten by his father for poor school performance. His mother was the patient who presented with symptoms of stress to my clinic and I tracked the cause to this underlying problem.’* (Diploma holder)

*‘ … identifying a perforated appendix in a child whose father requested a repeat prescription of an antispasmodic.’* (Diploma holder)

*‘I have learned to enquire about the family in my histories*.’ (Diploma holder)

Responders speak of taking ownership and an active interest in common clinical presentations in their practice, such as electing to engage in local investigation and clinical governance to improve their practice:

*‘I have learned to diagnose post-natal depression and I am now conducting an audit of this problem.’* (Diploma holder)

Some doctors disclosed their desire to engender further training in the wider clinical team, and take their new skills and knowledge forward to cascade their learning at a local level:

*‘I have become a teacher in the team (nurses, midwives, pharmacists).’* (Diploma holder)

*‘We have held our first workshop and are planning to hold another.’* (Diploma holder)

*‘I learned how to develop, organise and hold a workshop*.’ (Diploma holder)

## Discussion

### Summary

The FM PG Diploma was designed and delivered in an attempt to improve patient care in a resource-poor and politically unstable region beset by a higher than average burden of disease and risk factors. This evaluation demonstrates real gains in doctor–patient interaction in terms of the patient experience, and self-reported improvements in skills and abilities in participating doctors. Whether the improvements are sustainable in the long term remains to be seen, but in a country affected by illness, war, and poverty, even short-term gains are a small victory that merit dissemination. Though physicians with the Diploma benefited from this intervention, it may be difficult to implement a patient-centred and a biopsychosocial approach if physicians are not given adequate time in each consultation. UNRWA is engaged in improving the resources and infrastructure of their medical clinics, and the benefits of such engagement are the subject of a further article (in preparation).

### Strengths and limitations

The authors acknowledge that the small numbers of physicians and patients included in this evaluation limit the generalisability of the findings. The findings presented here are, in effect, drawn from a pilot intervention. Evaluation of successive years of graduates will generate richer data that will allow elaboration and refinement of the conclusions of this article. Statistical analysis did not take clustering into account; in other words, there were not sufficient numbers of doctors to correlate patient responses for the same doctor. This is another reason to be cautious in the interpretation of the data.

The absence of pre-training assessment of practitioners limits the ability to state with absolute certainty that the difference in patient satisfaction is solely due to the training involved in the Diploma. There is the possibility of bias due to the selection process of the Diploma training: those selected for the Diploma achieved the highest marks in the application test. This could explain some of the observed differences rather than the training programme. It should be noted, however, that academic achievement does not equate to excellent doctors, and the physician and patient feedback focused heavily on improved non-technical facets of health care, which were not assessed in the application test.

### Comparison with existing literature

The WHO Astana Declaration on primary health care in October 2018 specifically highlights the needs for training primary care professionals,^10^ but this is sometimes easier said than done. Medical education in resource-poor environments is fraught with difficulties.^11^ Previous educational programmes have identified challenges in recruiting and training local faculty, and establishing leadership;^12,13^ inadequate scale up; and lack of coordination.^14^ To establish successful programmes, there must be a coordinated effort between government policymakers and an alignment to educational institutions.^15^ The FM PG Diploma in Gaza is aligned to an established teaching university in the UK and is truly scalable; there is little difference between training 15 doctors and 150, due to the blended online platforms. The government in Gaza is fractured and unable to develop cohesive policies, but UNRWA provides stable local leadership.

Increasing morale was not a primary aim of this study, but analysis of the focus group discussions reveals a new enthusiasm for work. Learners speak of starting local training programmes and instigating audits. The diplomates have started their own social media group for ongoing dialogue and clinical needs, and set up regular journal club and case presentation sessions. Workforce morale is an important factor in the complex phenomenon of psychological resilience.^16,17^ The Middle East has moderate-to-high levels of self-reported burnout among healthcare workers,^18^ although the degree of burnout in Palestinian doctors is unstudied and unknown. Additionally, improved healthcare morale is linked to improved patient-reported care outcomes and patient experience.^19^

### Implications for research and practice

The e-learning platform and the blended form of delivery permits a scalable model, which can train hundreds of doctors as several cohorts every year. For example, 4 cohorts of 25 doctors would equate to 100 doctors per year, but larger cohorts would increase this number dramatically. Importantly, this model keeps costs low, and allows for scaling up in number of learners without massive increases in expenditure.

The synthesis of online and face-to-face teaching methods facilitates on-the-job learning, and minimises disruptions to the ongoing delivery of healthcare services. If this education model were to be adopted elsewhere, it is most likely to be in areas that require urgent postgraduate training but cannot afford to reduce its workforce to do so.

This exploratory study suggests that the postgraduate family medicine educational intervention has improved both the physicians’ and patients’ healthcare experience in an area suffering significant political and economic turmoil, minimal resources, and limited training opportunities. This education model is reproducible in other areas experiencing similar adversities. The programme is scalable, meaning that, without any significant changes in its core material and teaching approaches, the number of participating doctors can be vastly increased, potentially up-skilling scores or hundreds of family doctors every year without removing them from clinical practice. This model of training is practical, low cost, and effective, and many low- and middle-income countries could strengthen their family medicine workforce through such a training programme. A larger and more definitive evaluation is required in order to be more confident and precise in measuring the positive outcomes for this intervention.
